# Prognostic model and immunotherapy prediction based on molecular chaperone-related lncRNAs in lung adenocarcinoma

**DOI:** 10.3389/fgene.2022.975905

**Published:** 2022-10-13

**Authors:** Yue Xu, Tao Tao, Shi Li, Shuzhen Tan, Haiyan Liu, Xiao Zhu

**Affiliations:** ^1^ Marine Medical Research Institute, Guangdong Medical University, Zhanjiang, China; ^2^ Department of Gastroscope, Zibo Central Hospital, Zibo, China; ^3^ Guangdong Provincial Key Laboratory of Systems Biology and Synthetic Biology for Urogenital Tumors, Shenzhen Key Laboratory of Genitourinary Tumor, Department of Urology, The First Affiliated Hospital of Shenzhen University, Shenzhen Second People’s Hospital (Shenzhen Institute of Translational Medicine), Shenzhen, China; ^4^ Department of Dermatology, The First Affiliated Hospital of Guangzhou Medical University, Guangzhou, China; ^5^ Department of Cardiovascular Medicine, Nanchong Central Hospital, The Affiliated Nanchong Central Hospital of North Sichuan Medical College, Nanchong, China; ^6^ Laboratory of Molecular Diagnosis, The First Affiliated Hospital of Chongqing Medical University, Chongqing, China

**Keywords:** TCGA, LUAD, lncRNA, molecular chaperone-related lncRNA index, prognosis, immunotherapy

## Abstract

**Introduction:** Molecular chaperones and long non-coding RNAs (lncRNAs) have been confirmed to be closely related to the occurrence and development of tumors, especially lung cancer. Our study aimed to construct a kind of molecular chaperone-related long non-coding RNAs (MCRLncs) marker to accurately predict the prognosis of lung adenocarcinoma (LUAD) patients and find new immunotherapy targets.

**Methods:** In this study, we acquired molecular chaperone genes from two databases, Genecards and molecular signatures database (MsigDB). And then, we downloaded transcriptome data, clinical data, and mutation information of LUAD patients through the Cancer Genome Atlas (TCGA). MCRLncs were determined by Spearman correlation analysis. We used univariate, least absolute shrinkage and selection operator (LASSO) and multivariate Cox regression analysis to construct risk models. Kaplan-meier (KM) analysis was used to understand the difference in survival between high and low-risk groups. Nomogram, calibration curve, concordance index (C-index) curve, and receiver operating characteristic (ROC) curve were used to evaluate the accuracy of the risk model prediction. In addition, we used gene ontology (GO) enrichment analysis and kyoto encyclopedia of genes and genomes (KEGG) enrichment analyses to explore the potential biological functions of MCRLncs. Immune microenvironmental landscapes were constructed by using single-sample gene set enrichment analysis (ssGSEA), tumor immune dysfunction and exclusion (TIDE) algorithm, “pRRophetic” R package, and “IMvigor210” dataset. The stem cell index based on mRNAsi expression was used to further evaluate the patient’s prognosis.

**Results:** Sixteen MCRLncs were identified as independent prognostic indicators in patients with LUAD. Patients in the high-risk group had significantly worse overall survival (OS). ROC curve suggested that the prognostic features of MCRLncs had a good predictive ability for OS. Immune system activation was more pronounced in the high-risk group. Prognostic features of the high-risk group were strongly associated with exclusion and cancer-associated fibroblasts (CAF). According to this prognostic model, a total of 15 potential chemotherapeutic agents were screened for the treatment of LUAD. Immunotherapy analysis showed that the selected chemotherapeutic drugs had potential application value. Stem cell index mRNAsi correlates with prognosis in patients with LUAD.

**Conclusion:** Our study established a kind of novel MCRLncs marker that can effectively predict OS in LUAD patients and provided a new model for the application of immunotherapy in clinical practice.

## Introduction

Lung cancer is a malignant tumor originating from the bronchi and alveoli. Worldwide, 1.77 million lung cancer deaths occur each year, and it is the leading cause of cancer death in the world (Siegel et al., 2021). LUAD is a subtype of lung cancer and a highly heterogeneous malignancy, accounting for approximately half of all lung cancers (Sivakumar et al., 2017; Xu et al., 2020a). Studies have shown that the risk factors for LUAD mainly come from direct exposure to tobacco. LUAD tends to occur early in East Asian women who do not smoke (Chen et al., 2020; Devarakonda et al., 2021). This is related to the presence of Epidermal growth factor receptor (EGFR) mutations in East Asian LUAD patients (Choong and Sung, 2021; He et al., 2021). Lung cancer usually involves pleura and has a poor prognosis. The 5-year survival rate is less than 20% (Zhang et al., 2021). LUAD is prone to distant metastasis, and the common sites of metastasis are brain, liver, bone, adrenal gland and pleura (Klikovits et al., 2018). In the past few decades, treatment of LUAD has mainly included surgery, chemotherapy, and emerging immunotherapies. Although recent advances in LUAD have greatly improved the prognosis of LUAD patients, the OS of advanced LUAD patients is still very low. Therefore, developing new biomarkers to predict the prognosis of LUAD patients and find potential therapeutic targets for LUAD is crucial.

Molecular chaperones are molecular assistants that assist in the folding and assembly of intracellular proteins and play an important role in intracellular life activities (Shan et al., 2020; Wei et al., 2022). Studies have shown that molecular chaperones play an important role in the occurrence and development of tumors, and it has also been confirmed as a prognostic marker for tumors (Zhu et al., 2013; Lu et al., 2020). Jia *et al.* (Jia et al., 2021) found that heat shock protein 90 (HSP90) can promote the metastasis of LUAD cells by interacting with the oncogene EEF1A2. It ultimately leads to the poor prognosis of LUAD patients. In addition, drugs targeting molecular chaperone-related genes are also widely used in clinical practice. chaperones-related related genes with great clinical potential, such as HSP90 and P53, making immunotherapy become an important means of tumor treatment (Armstrong et al., 2018; Kaida and Iwakuma, 2021). Although molecular chaperones can be used as prognostic indicators in patients with LUAD, the utility of using only a single biomarker is limited. Therefore, establishing reliable biomarkers for the construction of LUAD prognostic models is an urgent clinical task.

LncRNAs refer to a class of RNAs longer than 200 nucleotides that cannot encode complete proteins (Vollmers and Carpenter, 2022). Studies have shown that lncRNAs play a crucial role in the occurrence and development of tumors, including LUAD (Dong et al., 2018). Meanwhile, studies have reported that immune infiltrating cells play a crucial role in the progression and invasion of tumor cells (Li et al., 2020a). LncRNA is a key regulatory element in the immune system, which has the functions of antigen presentation, antigen release, immune migration, immune infiltration, and immune activation (Xia et al., 2020; Zhang et al., 2020). With the deepening of research, the role of lncRNAs as ideal diagnostic markers for tumors has been gradually discovered (Peng et al., 2016). These all suggest that lncRNAs may be used as a new biomarker to improve the prognosis and treatment of LUAD patients. Zhou *et al.* (Zhou et al., 2022) found that lncRNAs can promote the occurrence and development of LUAD by binding to HSP90. However, the current research on the pathogenesis of MCRLncs in LUAD is still lacking. Therefore, we attempted to use transcriptome data and clinical data from the TCGA database to develop a marker of MCRLncs with guiding significance for immunotherapy. This study provides a new model with prognostic value for LUAD patients and establishes a new feature to predict LUAD patients’ response to immunotherapy.

## Materials and methods

### The data source

The Genecards is a comprehensive bioinformatics database from 125 web sources such as NCBI and UCSC, which provides detailed information on all currently annotated and predictable genes. It covers information including genome, proteome, transcription, and function (Safran et al., 2010; Stelzer et al., 2011). Through the Genecards database portal (https://www.genecards.org/), we obtained 233 genes related to chaperones (Relevance score ≥ 5). MSigDB is a treasure trove database for analyzing gene enrichment pathways (Liberzon et al., 2011). We downloaded 17 GSEA functional pathways from MsigDB (Supplementary Table S1). These pathways enriched 517 genes in total, and we removed duplicated genes to get 312 genes. Finally, we pooled the 233 genes obtained based on the Genecards database and the 312 genes obtained based on the MsigDB, removed the duplicate genes, and finally obtained 417 molecular-chaperone genes.

TCGA is a landmark Human Cancer Genome Project that has collected molecular numbers from more than 20,000 primary cancer samples, including LUAD (Cai et al., 2021a). In this study, we from the TCGA data portal website (https://portal.gdc.cancer.gov/) downloaded transcriptome data, clinical data, and mutation data in LUAD. Clinical data included OS, survival status, age, sex, tumor grade, and tumor node metastasis (TNM) stage. In this study, the samples were randomly divided into a training subgroup (*n* = 330) and a validation subgroup (*n* = 164). The training subgroup was used to construct a molecular chaperone-related lncRNAs risk model. The validation subgroup and the whole group were used to validate the risk model. In addition, the IMvigor210 dataset, a cohort of atezolizumab (anti-PD-L1 monoclonal antibody) for the treatment of bladder cancer, was extracted to evaluate the response of MCRLncs markers to immunotherapy efficacy (Powles et al., 2014).

### Identification of molecular chaperone-related lncRNAs in patients with LUAD

We used the Spearman correlation analysis (| cor | > 0.4 and *p* < 0.001) to identify MCRLncs. The heatmap showed the expression correlation between molecular chaperone genes and lncRNAs.

### Molecular chaperones-related lncRNAs risk models were constructed

We divided all patients with lung adenocarcinoma into a training subgroup (*n* = 330) and a validation subgroup (*n* = 164). Data from the training subgroup were used to construct a prognostic model. We screened 301 MCRLncs by using univariate Cox regression analysis (*p* values less than 0.05 were considered significant). To narrow down the independent variables and avoid overfitting prognostic features, we performed the LASSO regression analysis on these MCRLncs (Meier et al., 2008). Next, we performed multivariate Cox regression analysis on the MCRLncs obtained by LASSO regression analysis, and finally screened 16 MCRLncs as candidate genes. The 16 candidate genes were used to construct prognostic models. To construct risk characteristics and calculate risk scores, the coefficients and expressive values of MCRLncs screened out from LASSO regression were used to calculate an individual risk score. The risk score represents a prognostic feature of chaperone-related lncRNAs, which helps us to distinguish high-risk LUAD patients from low-risk LUAD patients. Our risk score calculation formula is as follows:

Coef_i_ was the coefficient of lncRNA in LASSO regression. Coefficient xi is the expression value of selected MCRLncs (Li et al., 2020b; Li et al., 2020c; Liu et al., 2020; Liang et al., 2021). This formula is used to calculate the risk score.

To build a more intuitive model, we divided LUAD patients into high-risk and low-risk groups using the median risk score as a cutoff point. Next, we plotted KM survival curves, risk score distribution maps, and heatmaps to identify differences in the expression of MCRLncs between high- and low-risk groups.

### Validation of risk prognostic models

We plotted the ROC curve and C-index curve to verify the prognostic value of our constructed prognostic model. Based on the results of multivariate Cox regression analysis, we constructed a nomogram that can predict the occurrence of 1-, 3-, and 5-year OS survival in patients with LUAD. Nomogram is widely used for graphical calculations of complex formulas with practical accuracy. We can obtain the score of each clinical feature from the nomogram and predict the 1-year, 3-year, and 5-year survival rates of LUAD patients through the total score. Next, we evaluated the performance of the nomogram by drawing a calibration curve.

### Correlation analysis of risk score and clinicopathological features

To further validate the accuracy and specificity of the prognostic model, we used univariate and multivariate Cox analyses to screen variables and further explore independent risk factors associated with LUAD prognosis. We mapped two forests based on independent prognostic analyses to determine whether the prognostic model can be used as an independent prognostic indicator without reference to other clinical characteristics, including age, sex, race, tumor grade, primary tumor (T), regional lymph nodes (N), distant metastasis (M), and risk score.

### Principal component analysis was used to assess high-risk and low-risk patients

To assess whether LUAD patients were discriminative between high and low-risk groups, we visualized gene expression profiles using dimensionality reduction techniques. The expression of coding genes, lncRNA genes, all genes and high-risk lncRNAs genes in the risk model was analyzed by PCA analysis.

### Go functional pathway and KEGG enrichment analysis of differential MCRLncs in LUAD

To understand the differential expression of MCRLncs between high and low-risk groups, we used the “limma” R package to perform differential expression analysis of lncRNAs in high and low-risk groups, and extracted differentially expressed genes (DEGs) for the risk model. Next, we performed GO functional annotation and KEGG pathway enrichment analysis on the significant DEGs using the “clusterProfiler” R package, and the false discovery rate (FDR) of 0.05 was considered statistically significant (Mazandu et al., 2017; Kanehisa et al., 2019; Zou et al., 2021; Liang et al., 2022). GO database standardized description of gene products from three levels of biological process (BP), cellular component (CC), and molecular function (MF). Through GO functional annotation, we can understand the biological functions and pathways of DEGs enrichment. KEGG enrichment analysis can know in which pathways DEGs are enriched.

### Immune infiltration analysis was based on the single-sample gene set enrichment analysis

In order to study the immune infiltration of individual samples from the high-risk group and the low-risk group, we first downloaded the expression levels of specific marker genes under 13 immune function pathways. Next, we calculated the enrichment score for 13 immune function pathways in each LUAD sample by using ssGSEA (Barbie et al., 2009). We used heatmaps to display the immune infiltration of prognostic lncRNAs in high-risk and low-risk groups.

### Calculation of tumor mutational burden

High tumor mutational burden (TMB) is defined as the total number of somatic gene coding errors, base substitutions, gene insertions or deletions detected per megabyte (Tang et al., 2019; Li et al., 2020d). Recent studies have shown that TMB is associated with OS after immunotherapy in multiple cancer types, and suggest that TMB can be used as a predictive biomarker of immune checkpoint inhibitor treatment response (Xu et al., 2020b; Lin et al., 2020; Tan et al., 2020). As suggested by the reviewer, we cited references to supplement the judgment that the higher the TMB, the better the outcome of tumor immunotherapy (Snyder et al., 2014). We performed TMB differential analysis for high-risk and low-risk groups, and combined TMB for KM analysis of both groups.

### Immune escape and immunotherapy analysis

The TIDE algorithm has been used in many articles to predict response to immunotherapy. TIDE data comes from the TIDE website (http://tide.dfci.harvard.edu/) (Fu et al., 2020). It is frequently used to predict response to immune checkpoint inhibitors such as the cluster of differentiation 274 (CD274) and cytotoxic T lymphocyte-associated antigen 4 (CTLA4) in both low-risk and high-risk groups (*p*-values < 0.05 were considered statistically significant). In this study, we used the TIDE scoring algorithm to predict tumor susceptibility to immune checkpoint inhibition, and then to evaluate the effect of immunotherapy. Immune checkpoints we used for prediction include TIDE, microsatellite instability (MSI) (Young et al., 1993; Bonneville et al., 2017), CAF (Sahai et al., 2020), tumor-associated macrophages M2 (TAMM2) (Rey-Giraud et al., 2012), CD8, CD274, dysfunction, exclusion (Nishino et al., 2017; Wang et al., 2019a), myeloid-derived suppressor cell (MDSC) (Gabrilovich, 2017), Merck18 and interferon-G (IFN-G) (Nishino et al., 2017). A lower immune cell proportion score (IPS) indicates a good response to immunotherapy. Comprehensive scoring of immune checkpoints can help us identify the role of lncRNAs in tumor immune escape in high- and low-risk groups, and further predict the effects of immunotherapy models.

### Chemical drug sensitivity prediction

To explore the sensitivity of each LUAD patient from the TCGA database to different chemotherapeutic agents, we used the “pRRophetic” R package to identify potential therapeutic agents for LUAD patients. In the results, we used IC50 to represent the sensitivity of each patient to different chemotherapeutics (Zhu et al., 2012; Yang et al., 2021; Lai et al., 2022). High-risk and low-risk groups were used for comparison. The “pRRophetic” software package, which has been widely used in clinical studies of tumors, predicts IC50 by creating a statistical model based on drug sensitivity and RNA-seq data from genomics of drug sensitivity in cancer (GDSC) (www.cancerrxgene.org/) (Cai et al., 2021b).

### Stem cell index mRNAsi in LUAD patients and its clinical significance

Stemness indices, which describes the similarity of tumor cells to stem cells, can be used as prognostic indicators to help predict the risk of tumor recurrence and guide treatment (Malta et al., 2018). MRNAsi is indices calculated based on gene expression data. We merged clinical data and stem cell data from LUAD and performed survival analysis on the merged data by using the “survival” R package. Meanwhile, we used the Wilcoxon test to investigate whether the stem cell index was correlated with age, gender, T, and M stage of LUAD patients.

### Statistical analysis

We performed all statistical analyses using R software (version 4.1.3). Target genes were screened using the Cox regression algorithm, a risk prediction model was established and a risk score was calculated. The prognostic value of the risk scoring model was assessed using the ROC curve, and the area under the curve (AUC) was calculated using the “timeROC” R package. In addition, we divided patients into high-risk and low-risk groups based on risk scores. KM analysis was used to compare differences in survival between patients with high and low-risk groups and to analyze differences in survival between different subgroups. Finally, we performed univariate and multivariate Cox regression analyses to identify independent prognostic factors for LUAD.

## Results

**FIGURE 1:**
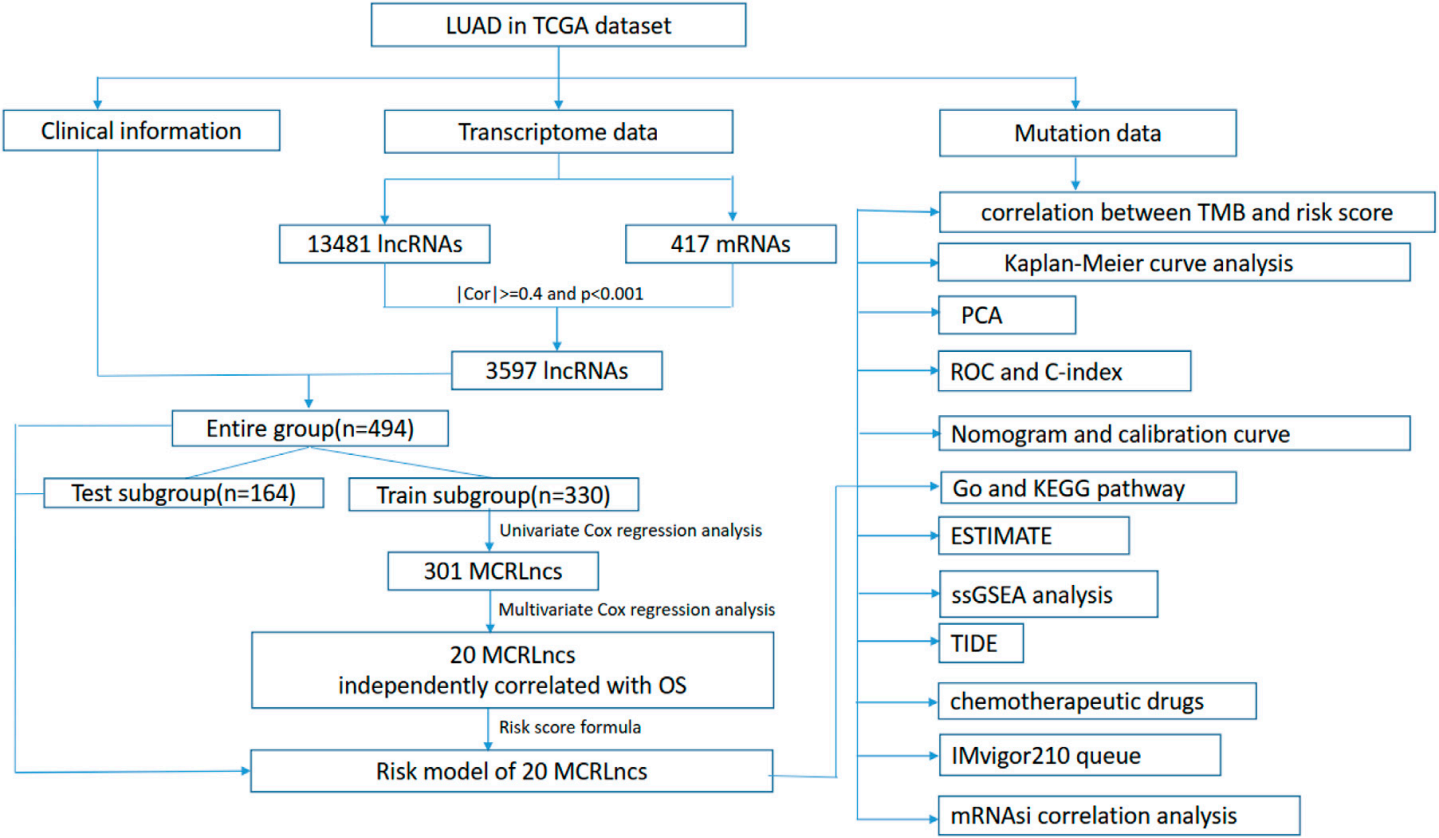
Flow chart of this study.

The flowchart of the present study was summarized in Figure 1.

### Identification of molecular chaperone-related lncRNAs in LUAD patients

**FIGURE 2:**
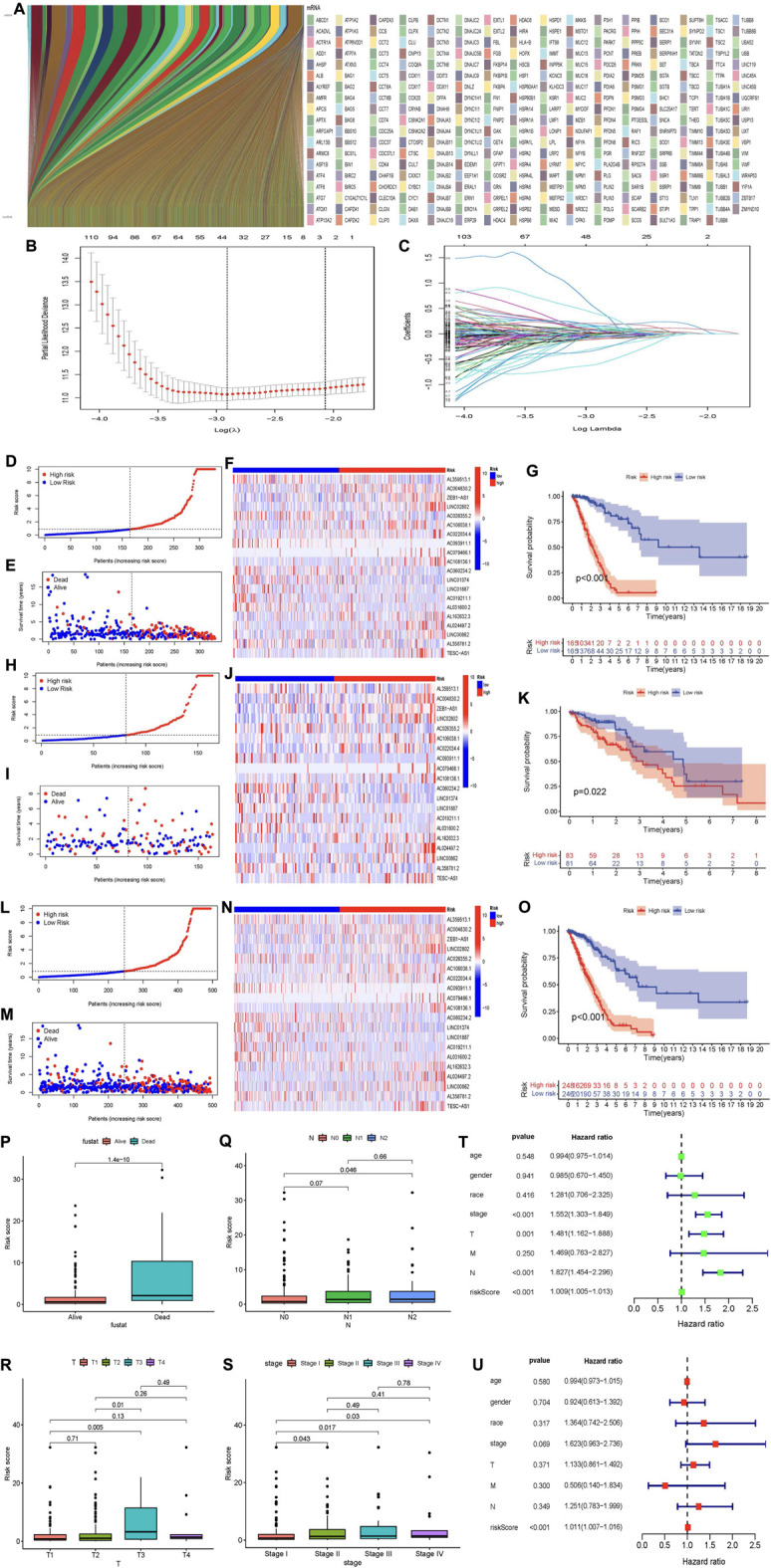
(Continued).

We identified a total of 3,597 MCRLncs by Spearman correlation analysis (|cor| > 0.4 and *p* < 0.001). We also mapped the molecular chaperone-lncRNAs co-expression network (Figure 2A). At the same time, in order to reflect the correlation between molecular chaperones and lncRNAs, we visualized the relationship between the two in a heatmap manner (Supplementary Figure S1). Finally, we merged the co-expressed lncRNAs with clinical information from patients with LUAD.

Risk signatures based on molecular chaperone-related lncRNAs were associated with prognosis in patients with LUAD.

We screened 301 MCRLncs by using univariate Cox regression analysis. LASSO regression analysis further screened 330 MCRLncs (Figures 2B,C). Multivariate Cox regression analysis showed that 16 MCRLncs were significant (*p* < 0.05) (Supplementary Table S2). The 16 prognostic MCRLncs were AL359513.1, AC004830.2, ‘ZEB1-AS1′, LINC02802, AC026355.2, AC106038.1, AC022034.4, AC093911.1, AC079466.1, AC108136.1, LINC01887, AC019211.1, AL031600.2, AL162632.3, AL024497.2 and LINC00862. In addition, we randomly divided the whole LUAD samples into two groups, the training subgroup (*n* = 330) and the validation subgroup (*n* = 164). To verify that the grouping was reasonable, we generated a clinical file by multivariate Cox regression analysis. The results showed that the *p* values of the clinical information between the two groups were all greater than 0.05, which indicated that there was no statistical significance in each clinical index between the two groups, that is, the grouping was good, and the statistical deviation error could be avoided (Table 1).

**TABLE 1:** Clinical data of training subgroup, validation subgroup and whole group.

Covariates	Type	Total *n* = 494	Test Subgroup *n* = 164<	Train Subgroup *n* = 330	*p*-Value
fustat	Alive	190(64.63%)	63(62.38%)	127(65.8%)	0.649
	Dead	104(35.37%)	38(37.62%)	66(34.2%)	
age	≤65	147(50%)	53(52.48%)	94(48.7%)	0.6233
	>65	147(50%)	48(47.52%)	99(51.3%)	
gender	FEMALE	155(52.72%)	54(53.47%)	101(52.33%)	0.9506
	MALE	139(47.28%)	47(46.53%)	92(47.67%)	
race	AMERICAN INDIAN OR ALASKA NATIVE	1(0.34%)	1(0.99%)	0(0%)	0.4592
	ASIAN	5(1.7%)	2(1.98%)	3(1.55%)	
	BLACK OR AFRICAN AMERICAN	27(9.18%)	11(10.89%)	16(8.29%)	
	WHITE	261(88.78%)	87(86.14%)	174(90.16%)	
stage	Stage I	153(52.04%)	49(48.51%)	104(53.89%)	0.5155
	Stage II	72(24.49%)	30(29.7%)	42(21.76%)	
	Stage III	51(17.35%)	16(15.84%)	35(18.13%)	
	Stage IV	18(6.12%)	6(5.94%)	12(6.22%)	
T	T1	96(32.65%)	35(34.65%)	61(31.61%)	0.644
	T2	162(55.1%)	57(56.44%)	105(54.4%)	
	T3	23(7.82%)	6(5.94%)	17(8.81%)	
	T4	13(4.42%)	3(2.97%)	10(5.18%)	
M	M0	276(93.88%)	95(94.06%)	181(93.78%)	1
	M1	18(6.12%)	6(5.94%)	12(6.22%)	
N	N0	189(64.29%)	63(62.38%)	126(65.28%)	0.7674
	N1	60(20.41%)	23(22.77%)	37(19.17%)	
	N2	45(15.31%)	15(14.85%)	30(15.54%)	

Based on the median risk score, we divided LUAD patients into high-risk and low-risk groups. We performed survival analysis on LUAD samples using the “survival” package of R software and plotted survival curves. The distribution of risk scores in the low-risk group and the high-risk group is shown in Figure 2D. The survival status and survival time of patients in the two different risk groups are shown in Figure 2E. The relative expression criteria of MCRLncs for each patient is shown in Figure 2F. KM survival analysis showed that patients in the low-risk group lived longer than those in the high-risk group (*p* < 0.001) (Figure 2G). The distribution of risk classes, the corresponding survival status, and the relative expression levels of the 16 MCRLncs suggested that high-risk indices are associated with high mortality (Figures 2D–F). At the same time, we found that lncRNAs LINC02802, AC022034.4, AC079466.1, AC108136.1 and AL024497.2 were significantly associated with a higher risk of tumor death (Figure 2F). We also found that LINC01887, AC019211.1 and AL031600.2 were highly expressed in the high-risk subgroup and low expressed in the low-risk subgroup. To test the prognostic power of this risk model, we calculated a risk score for each patient in the validation and whole groups using a unified risk formula. Figures 2H–K, 2L-O represent the distribution of risk score, survival status and survival time, and the expression of MCRLncs in the validation subgroup and the whole group, respectively. KM survival analysis between the validation subgroup and the whole group showed no difference in the TCGA group, and patients in the high-risk group had lower OS than those in the low-risk group (Figures 2K,O). This indicates that the prognostic model of LUAD constructed by us is effective.

### Clinical assessment using risk assessment models

We used the “ggpubr” R package to perform a correlation analysis of clinical information and risk score. Figure 2P–S shows that the fustat, T stage, regional lymph nodes (N) stage, and tumor grade are significantly correlated with the calculated risk score. We found an increased risk of death from stage I to stage III (*p* < 0.05) (Figure 2S). Patients who died had higher risk scores (*p* < 0.05) (Figure 2P).

Next, we performed univariate and multivariate Cox analyses to identify prognostic factors in patients with LUAD (Supplementary Table S3). Univariate Cox analysis showed tumor grade stage (*p* < 0.001, Hazard ratio (HR) = 1.552, 95% confidence interval (95%CI): [1.303–1.849]), T stage (*p* = 0.001, HR = 1.481, 95%CI: [1.162–1.888]), N stage (*p* < 0.001, HR = 1.827, 95%CI: [1.454–2.296]), Risk score (*p* < 0.001, HR = 1.009, 95%CI: [1.005–1.013]) showed statistical difference (Figure 2T). However, by multivariate Cox analysis only the risk value risk score (*p* < 0.001, HR = 1.011, 95%CI: [1.007–1.016]) showed a statistical difference. This just shows that risk score is closely related to the survival time of LUAD patients (Figure 2U).

### Evaluation and validation of risk prognostic models

**FIGURE 3:**
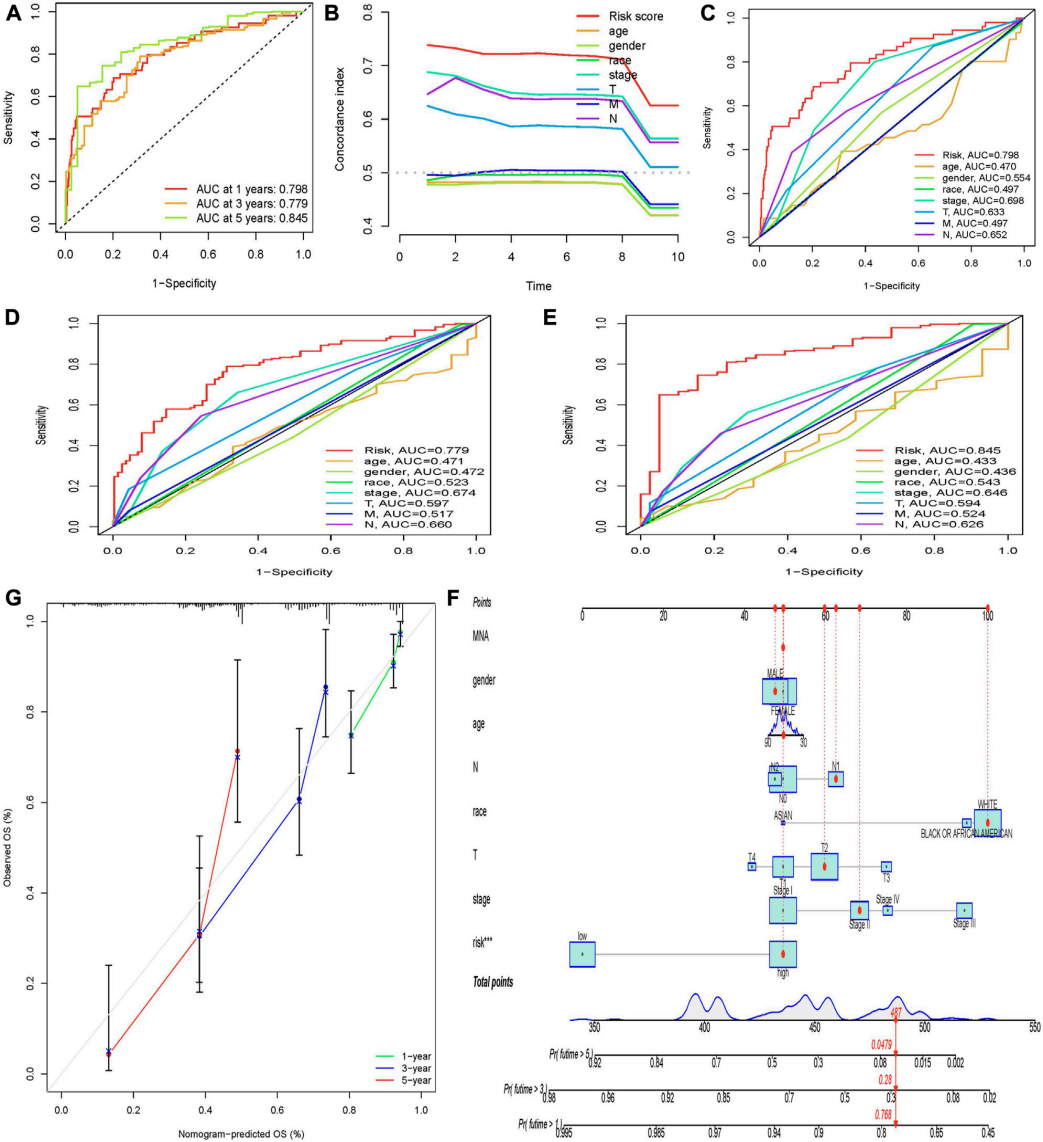
Evaluation of prognostic models and establishment of a nomogram. **(A)** AUC values of the prognostic model of molecular chaperones-related lncRNAs at 1, 3, and 5 years. The abscissa represents 1-specificity and the ordinate represents sensitivity. Interpretation of AUC results: AUC ≤0.5 indicates no predictive power. 0.5 < AUC ≤0.7 indicates low prediction accuracy. 0.7 < AUC ≤0.9 indicates moderate prediction accuracy. AUC >0.9 indicates high prediction accuracy. **(B)** C-index curve to assess the quality of a patient’s clinically independent prognostic model. **(C–E)** Calculate the AUC for risk score, age, gender, grade, and TNM stage of the total survival risk score according to 1-, 3-, and 5-year ROC curve. **(F)** Nomogram predicts 1-, 3-, and 5-year OS survival in patients with LUAD. **(G)** Calibration curve for nomogram. The *x*-axis is the nomogram predicted survival and the *y*-axis is the actual survival.

To evaluate the specificity and sensitivity of these prognostic factors, we evaluated our prognostic model using AUC and C-index. The 1-year, 3-year, and 5-year ROC curves showed that the AUC for predicting 1-year, 3-year, and 5-year survival rates of LUAD patients were 0.798, 0.779, and 0.845, respectively (Figure 3A). All the AUC were greater than 0.5, indicating that the predictive power of the prognostic model was credible. The results of the 5-year ROC curve showed that most of the clinical indicators we used could be used as predictors of 5-year survival in LUAD patients, except for age and sex. It is noteworthy that in the constructed prognostic model, the risk score was significantly better than other clinical variables in predicting 1-year, 3-year, and 5-year survival in LUAD patients (Figures 3C–E). Then, we used the C-index curve to further verify the prediction ability of the model. We found that the C-index value of risk score, stage, N, and T is greater than 0.5, indicating that the model has good prediction ability. Among them, the C-index of risk score was the largest and the prediction ability is the best (Figure 3B).

**FIGURE 4:**
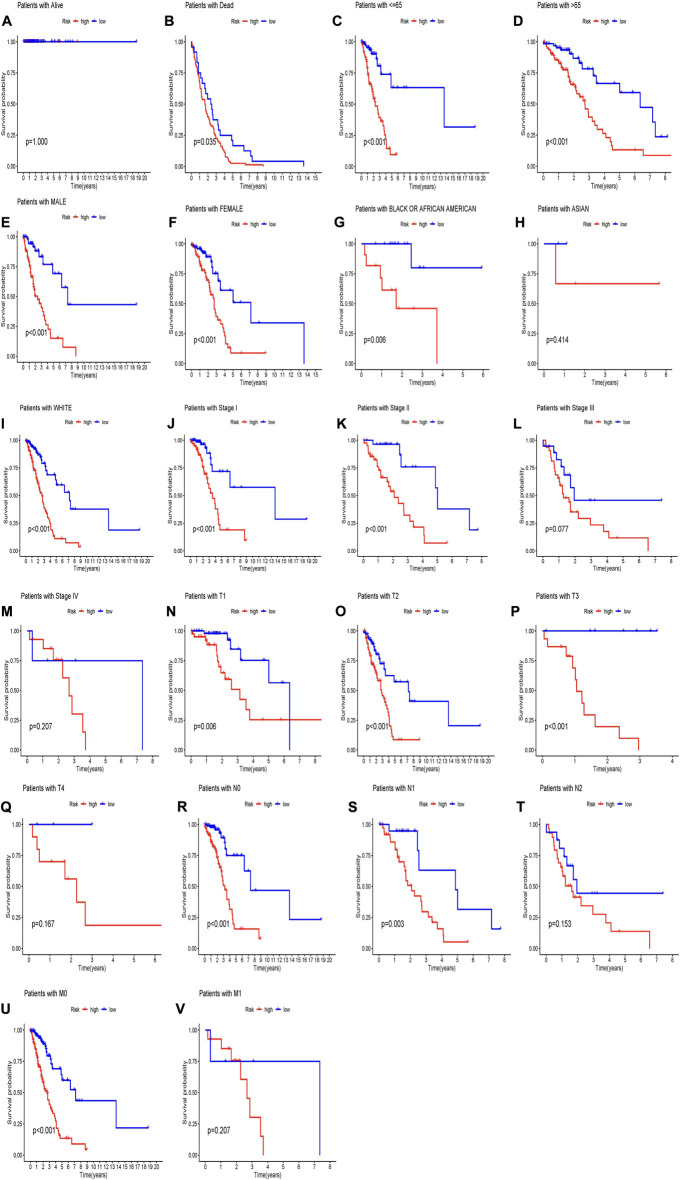
(Continued).

**FIGURE 5:**
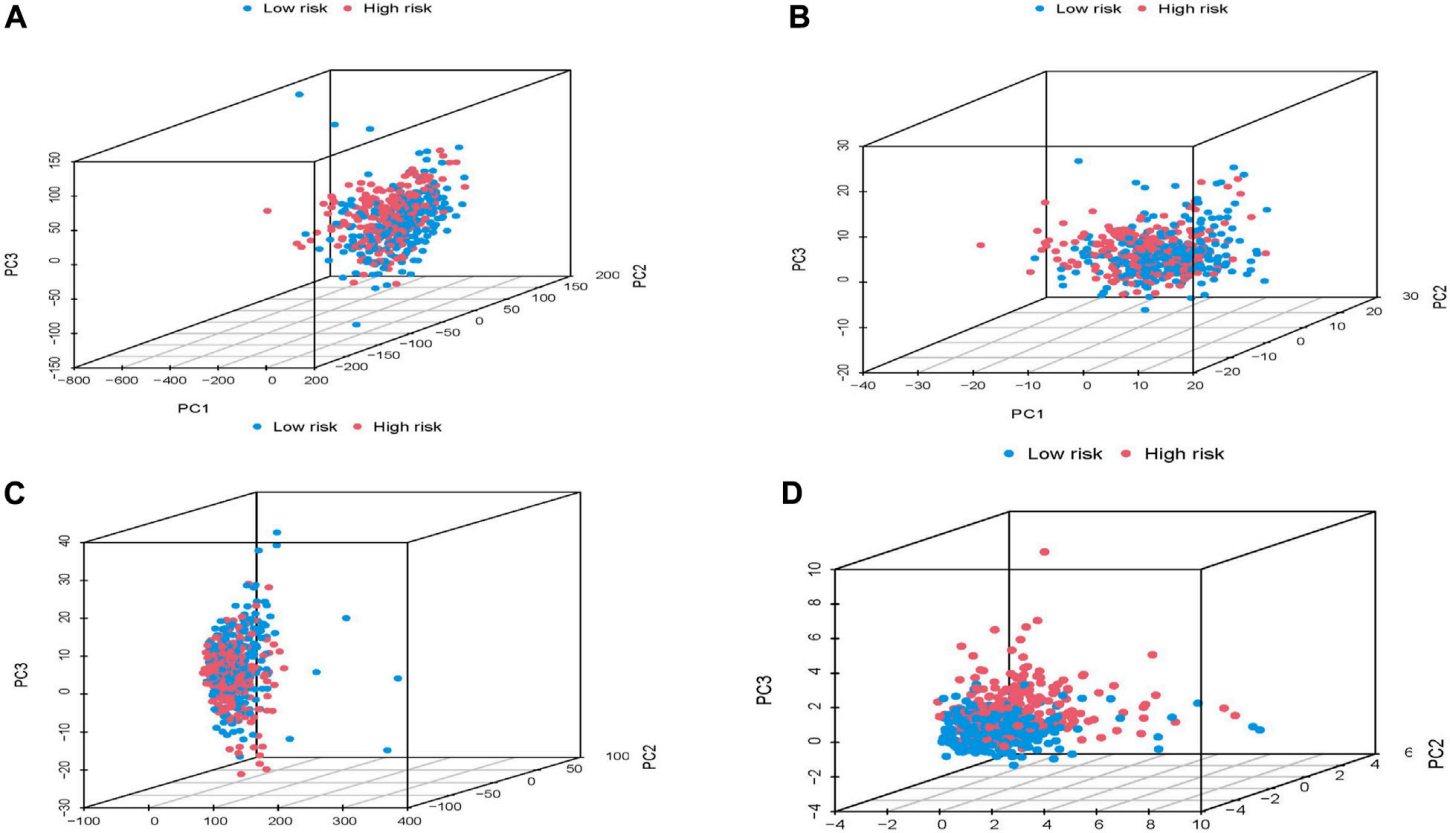
Principal component analysis of high and low risk groups based on TCGA whole group. **(A)** all genes. **(B)** 417 molecular chaperone genes. **(C)** 3,597 molecular chaperone-related lncRNAs. **(D)** 20 molecular chaperone-related lncRNAs in the risk model.

In order to predict the OS of patients with LUAD, we developed a nomogram widely used to predict the prognosis of cancer patients. The nomogram is based on two predictors, the risk score and associated clinical factors. We found that the nomogram was a good predictor of 1-, 3-, and 5-year survival in patients with LUAD (Figure 3F). This has certain significance for guiding clinicians to predict the survival time of LUAD patients. Further calibration curves showed that the 1-year and 3-year overall survival rates in the nomogram were consistent with the actual survival rates, indicating that the nomogram model can accurately predict the survival of patients with LUAD. Then, we performed KM survival analysis on LUAD patients grouped according to clinicopathological characteristics to validate our constructed clinically independent prognostic model. The results showed that patients in the high and low-risk groups had a shorter survival time over time, and patients in the high-risk group had a poorer prognosis, which was also expected (Figure 4B). We observed that compared with patients with low risk scores, male patients (*p* < 0.001), female patients (*p* < 0.001) (Figures 4E,F), patients younger than 65 years of age (*p* < 0.001), patients older than 65 years of age (*p* < 0.001) (Figures 4C,D), and black or African American patients (*p* = 0.006), White patients (*p* < 0.001) (Figures 4G,I), stage I patients (*p* < 0.001), stage II patients (*p* < 0.001) (Figures 4J,K), stage T1 patients (*p* = 0.006), stage T2 patients (*p* < 0.001), stage T3 patients (*p* < 0.001) (Figure 4–P), N0 stage patients (*p* < 0.001), N1 stage patients (*p* = 0.003), (Figure 4R, S), and M0 stage patients (*p* < 0.001) (Figure 4U). However, in the high-risk and low-risk groups, survival was not associated with risk scores for stage III (*p* = 0.077) and IV (*p* = 0.207) (Figure 4L, M), T4 (*p* = 0.167) (Figure 4Q), N2 (*p* = 0.153) (Figure 4T), and M1 (*p* = 0.207) (Figure 5). Asian patients were deleted due to insufficient data (Figure 4H).

In conclusion, patients in the low-risk group continued to survive longer than the high-risk group according to subgroups by survival status, age, ethnicity, and tumor grade. Therefore, after preliminary validation, it was shown that 16 MCRLncs markers we used to construct the risk model were closely related to clinical characteristics and could predict the survival time of LUAD patients.

### Use principal components analysis to verify the performance of risk models

We used PCA dimensionality reduction analysis to examine the different distribution of all gene expression profiles, 417 molecular chaperone gene expression profiles, 3,597 MCRLncs expression profiles, and 16 risk-related MCRLncs expression profiles (Figures 5A–D). The results showed that the patients in the high and low-risk groups divided by the 16 molecular chaperone-related lncRNAs in the risk model were obviously distributed in different directions. The distribution of patients divided by the other three methods is relatively scattered, which indicates that the constructed prognostic model is of great help in identifying patients in the high-risk group and the low-risk group, which just shows that there are significant differences in immunity between the two groups of patients.

### Functional characteristics of risk prognosis models

**FIGURE 6:**
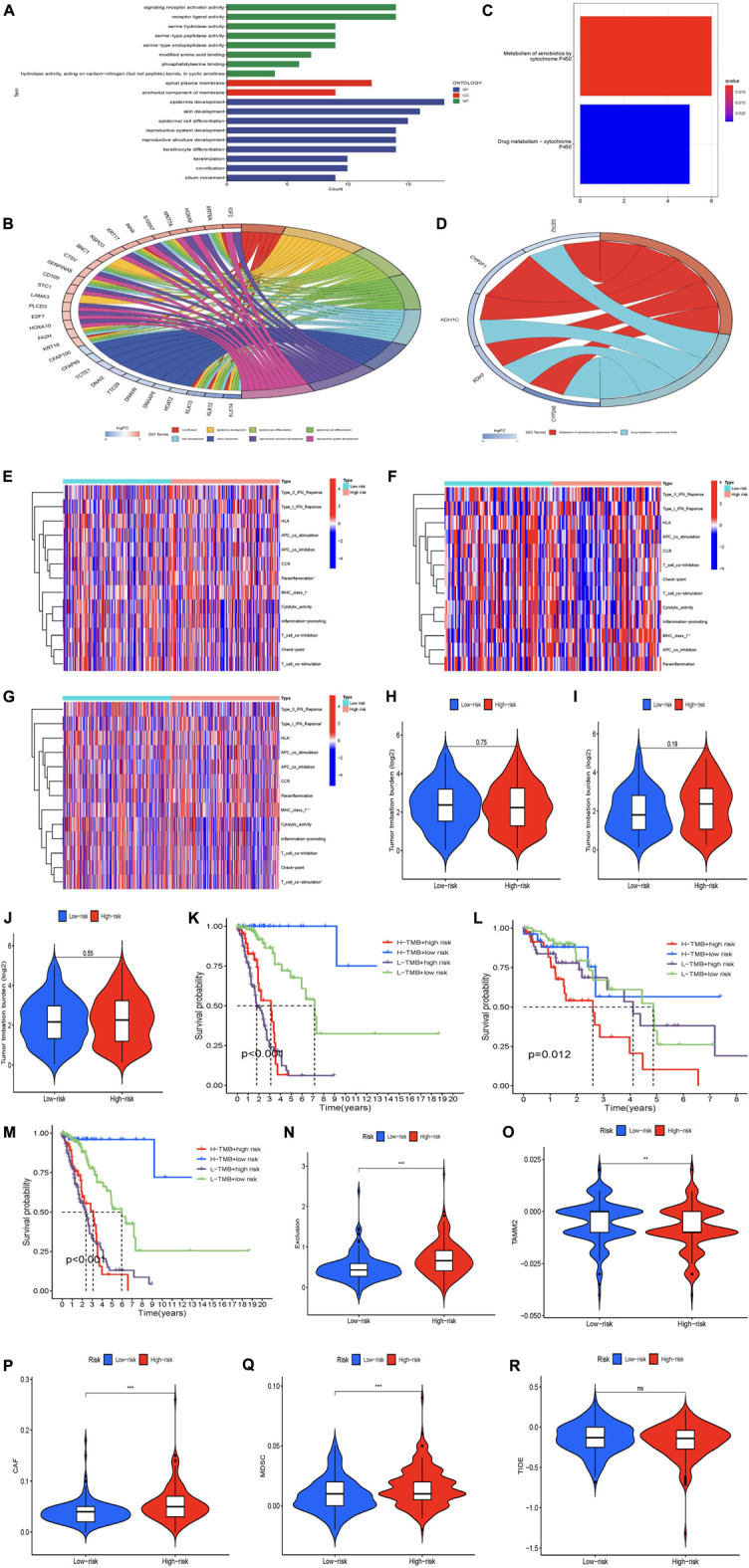
(Continued).

To further elucidate the potential biological functions and major signaling pathways of prognostic lncRNAs, we performed GO functional pathways and KEGG enrichment analysis (FDR <0.05). The results showed that the differential lncRNAs genes we studied played a role in regulating the malignant processes such as BP, CC, and MF in LUAD (Figure 6A). BP found that these genes were mainly enriched in epidermis development-related pathways. In terms of CC, these genes were mainly enriched in apical plasma membrane-related pathways. In terms of MF, these genes were mainly enriched in signaling receptor activator activity and receptor ligand activity related pathways. Figure 6B shows which functional pathways the differential lncRNAs are clustered in. We also found that lncRNA CFAP100, CFAP65, TCTE1, DNAI2, TTC29, DNAH9, DNAAF6, and HOATZ are all involved in the functional pathway of cilium movement (Figure 6B). Figures 6C,D shows that differential lncRNA genes were mainly enriched in two metabolic pathways, Metabolism of xenobiotics by cytochrome P450 and drug metabolism - cytochrome P450. We also found that lncRNA CYP2F1, CYP2A6, ADH1C, GSTA2, ADH7, and GSTA1 genes are all involved in regulating the metabolic pathway of Metabolism of xenobiotics by cytochrome P450 (Figure 6C). In conclusion, we found that many immune-related biological processes play a role in the risk assessment of chaperone-related lncRNA models.

### Risk models to assess the tumor immune microenvironment

Based on the results of functional enrichment, we speculate that the tumor immune microenvironment of LUAD patients may be related to MCRLncs. We used the “GSVA” R package to analyze the immune infiltration of lncRNAs in high and low-risk groups with 13 immune functions, and the results were presented in the form of heatmaps (Figures 6E–G). In the training subgroup, we found that lncRNAs were correlated with the two immune function pathways, Parainflammation and MHC_class_I, and were highly expressed in the high-risk group. In addition, the immune function pathway MHC_class_I was found to be correlated with lncRNAs in the training set, validation set, and all samples. This indicated that there were significant differences in the expression of lncRNAs in the low-risk group and the high-risk group in the expression of immune markers.

TMB is frequently used as a predictive biomarker for response to immune checkpoint inhibitor therapy. The results in Figures 6H–J show that high and low-risk groups were not associated with TMB. In the survival analysis, we found that the low-risk group with high TMB in blue had the highest probability of survival, followed by the low-risk group with low TMB in green. In contrast, both the high-risk purple group with low TMB and the high-risk red group with high TMB had poor survival probabilities (Figure 6K). Results in the validation group and the whole group were not statistically significant (Figure 6L, M). These results suggest that high TMB can be considered a protective factor in patients with LUAD. Although the relationship between the risk model and mutational burden was not clear, in the pooled survival analysis, the high-risk group with high TMB in red was significantly different from the other three groups. This is helpful for our prognosis.

Currently, immune checkpoint inhibition therapy is a promising modality for cancer treatment. We used the TIDE score to simulate tumor immune escape to predict the efficacy of immune checkpoint inhibitor therapy. Immunoassay results showed that TIDE scores were not statistically significant in high and low-risk groups (Figure 6R). The comprehensive score of exclusion and CAF is higher in the high-risk group, which suggests that the higher the score of exclusion and CAF, the lower the risk of tumor (Figure 6N, P). TAMM2 and MDSC were lower in the high-risk group, indicating that the lower the TAMM2 and MDSC score, the higher the risk of tumors, which contradicts the actual (Figure 6O, Q). Analysis of other immune checkpoints showed no statistical significance (Figure 6S–Z). Therefore, we speculate that the addition of Exclusion and CAF will promote the development of LUAD in high-risk groups.

### Prediction of chemosensitivity in patients with LUAD using risk scores

**FIGURE 7:**
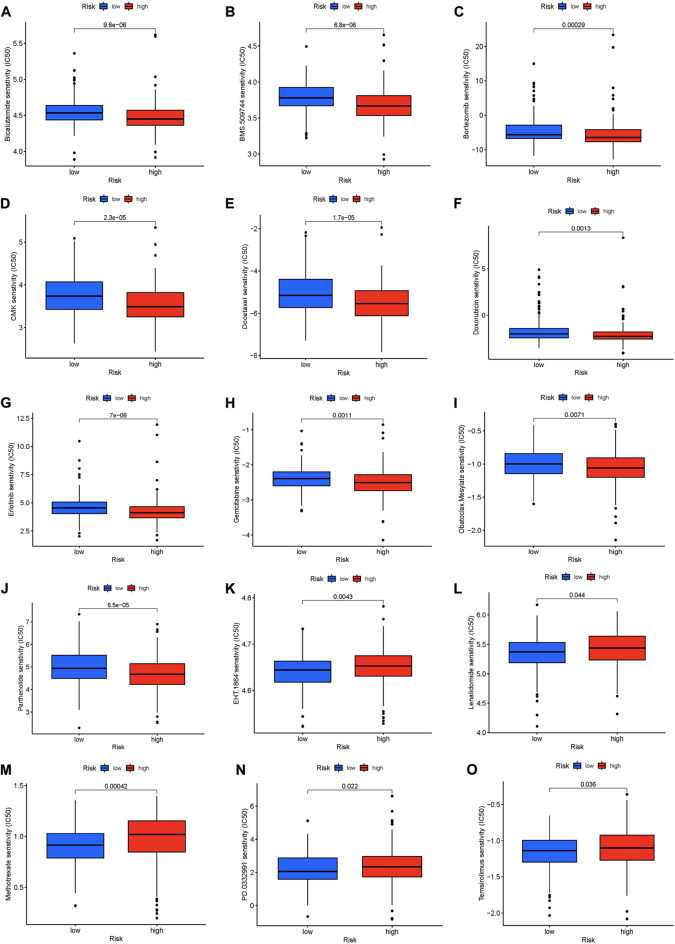
(Continued).

To analyze the prediction of LUAD patients’ response to chemotherapy agents, we used the “pRRophetic” R package to study the sensitivity of the high and low-risk groups to different chemotherapy agents. Figures 7A–J shows that, Bicalutamide, BMS.509,744, Bortezomib, CMK, Docetaxel, Doxorubicin, erlotinib, Gemcitabine, Obatoclax. The lower the semi-inhibitory concentration of mesylate and patlactone in the high-risk group, the higher the risk score. This suggests that these drugs are good for the treatment of LUAD patients in the high-risk group. Instead, we found that. EHT. 1864, Lenalidomide, Methotrexate, PD.0332991, and Temsirolimus had higher sensitivity in the low-risk group (Figure 7J–O). In conclusion, the analysis of chemotherapy sensitivity of LUAD patients can provide thoughts for the clinical treatment of LUAD patients.

### Immunotherapy model validation and immunotherapy response analysis

**FIGURE 8:**
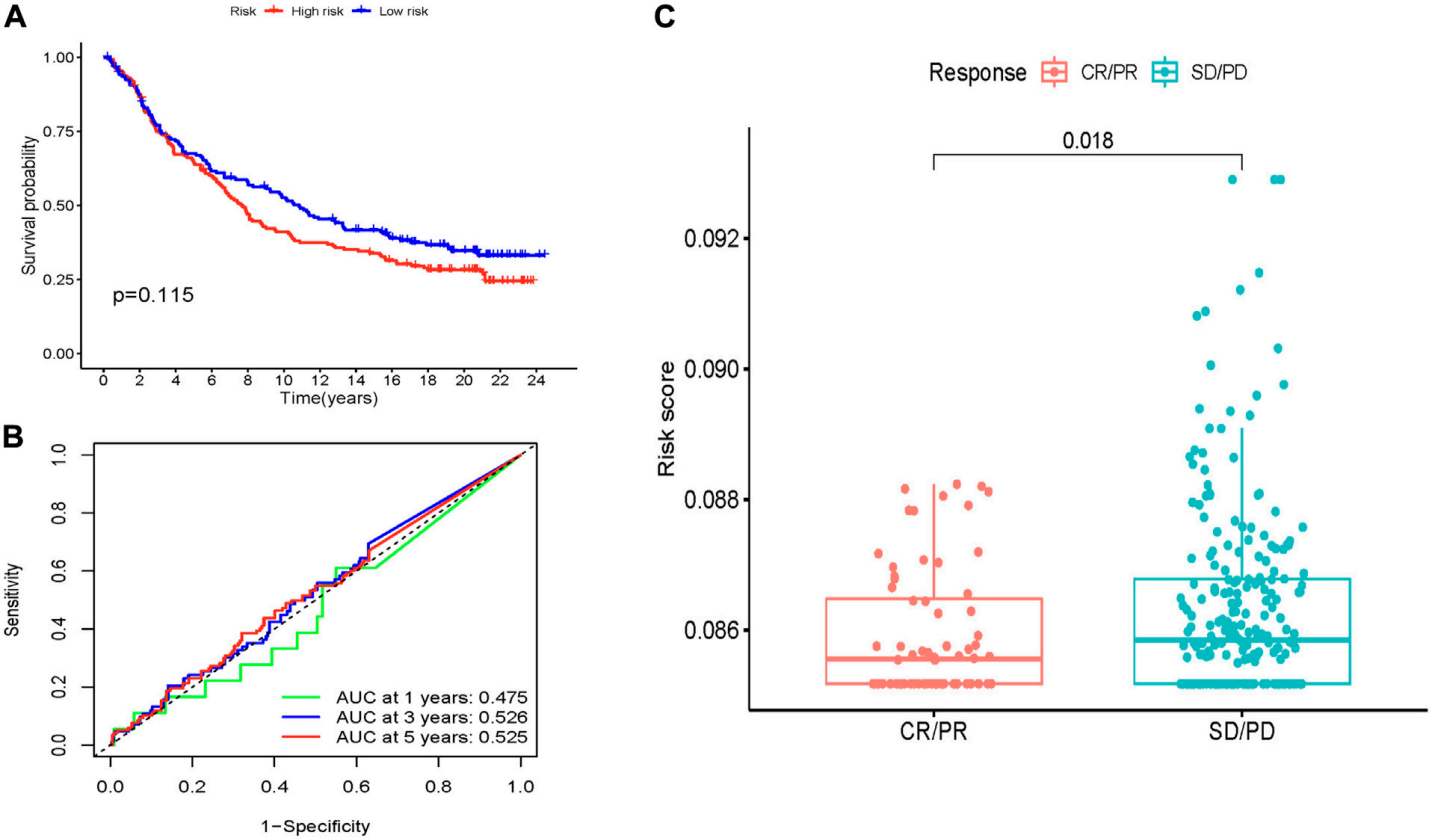
Predictive value of MCRLncs for immunotherapy response in the IMvigor210 cohort. **(A)** Kaplan-Meier estimates of overall survival for patients in the low-risk or high-risk groups suggest that survival in the low-risk group was not statistically significant. **(B)** In the IMvigor210 cohort, the AUC values of the molecular chaperones-related lncRNA prognostic model screened by Lasso regression at 1, 3 and 5 years were 0.475, 0.526 and 0.525, respectively. **(C)** Comparison of risk scores between partial response and progressive disease suggests that lower MCRLncs scores may predict better immunotherapy response.

To verify the reliability of the immunotherapy model we constructed, we analyzed the IMvigor210 cohort of bladder cancer patients enrolled in immunotherapy. KM survival analysis showed that when the target gene was expressed in the IMvigor210 cohort, the survival probability between high and low-risk groups was not statistically significant (*p* > 0.05) (Figure 8A). Next, we used the ROC curve to verify the immunotherapy model we constructed. Unfortunately, the ROC curve was not a good predictor (Figure 8B). The results of immunotherapy response analysis showed that the risk score of target genes in the IMvigor210 cohort was significantly different (*p* < 0.05), indicating that immunotherapy drugs have a good therapeutic effect on target genes (Figure 8C).

### Stem cell index mRNAsi and clinical features correlated with prognosis

**FIGURE 9:**
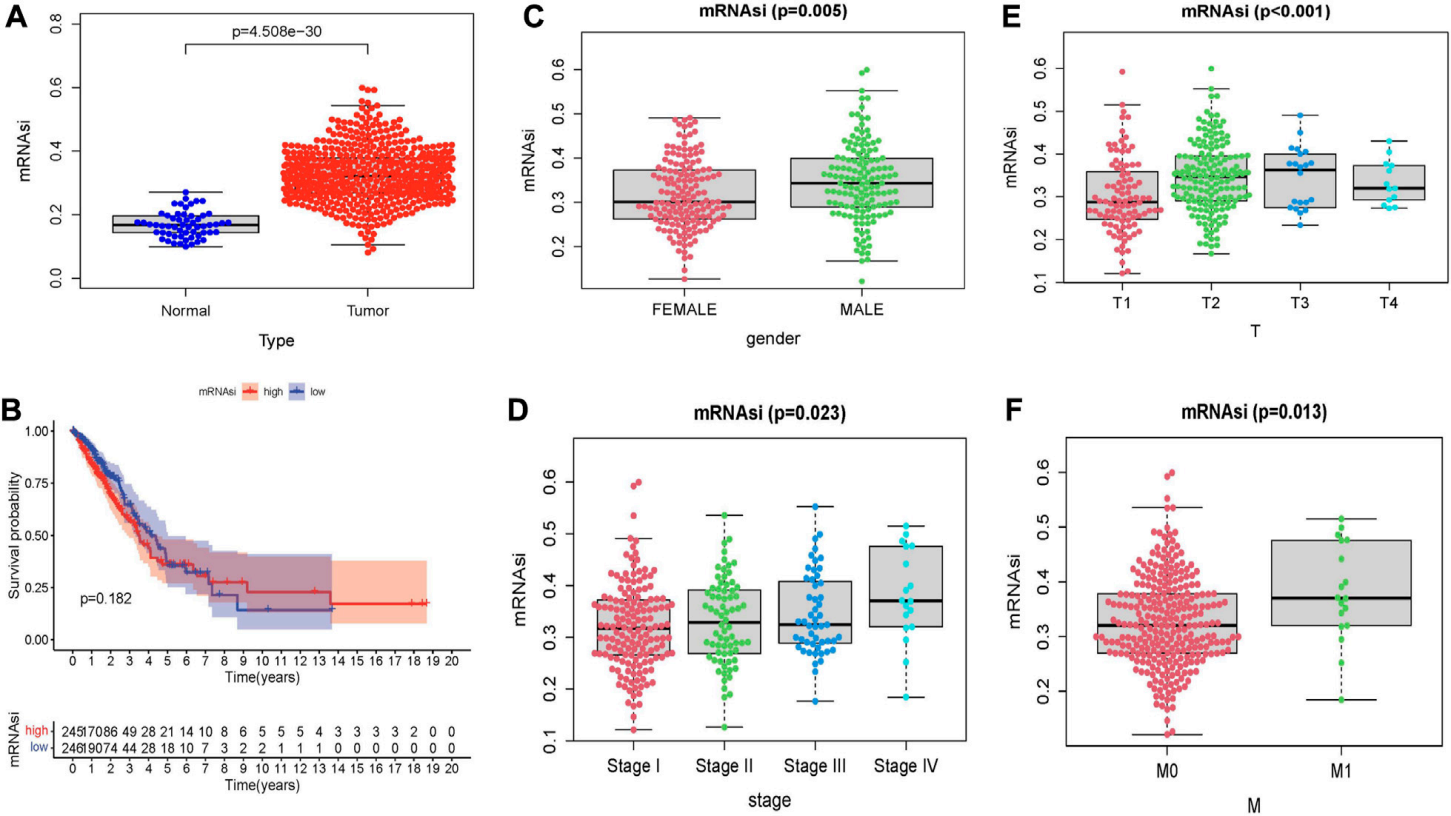
Relationship between stem cell index and prognosis and clinicopathological features in LUAD. **(A)** Differences in mRNAsi between normal and tumor tissues. **(B)** Kaplan–Meier survival curves of mRNAsi in LUAD. Comparison between mRNAsi expression level and clinical characteristics in LUAD, including **(C)** gender, **(D)** tumor grade, **(E)** T stage, **(F)** M stage.

By analyzing the stem cell index mRNAsi expressed in LUAD patients, the results showed that the mRNAsi of lung cancer samples and normal samples showed significant statistical differences (*p* < 0.05) (Figure 9A). KM survival analysis showed that there was no statistical significance in survival probability between high and low-risk groups (*p* > 0.05) (Figure 9B). Next, we explored the relationship between stem cell index and clinical indicators. We found significant differences in stem cell indexes of LUAD patients by gender, stage, and T and M stages (Figures 9C–F). This indicates that mRNAsi in LUAD patients is highly correlated with clinical indicators, and the study of stem cell index plays an important role in improving the prognosis of LUAD patients and developing new immunotherapy.

## Discussion

Lung cancer is a malignant tumor with the highest morbidity and mortality in China and the world, and the 5-year survival rate is less than 20% (Zhu et al., 2019; Siegel et al., 2022). The main risk factors for lung cancer are smoking and second-hand smoke. LUAD is the most important pathological type of lung cancer, accounting for about half of all lung cancers, and its incidence is gradually increasing in China. In recent years, a large number of studies have shown that tumor markers in LUAD have achieved positive outcomes in terms of prognosis and treatment (Liang et al., 2020; Song et al., 2021a). Despite progress in LUAD treatment, there are still a large number of LUAD patients who do not have appropriate treatment. This may be due to the fact that LUAD is a highly heterogeneous tumor and individual differences between LUAD patients are obvious. Therefore, there is an urgent need to construct a new prognostic model to accurately predict the prognosis of LUAD patients at an early stage and improve new immunotherapy targets.

Previous studies have shown that molecular chaperones can promote the development of lung adenocarcinoma. For example, Huang, Z. C. *et al.* (Huang et al., 2018) found through bioinformatics studies that high expression of HSPB1 could promote the growth of lung adenocarcinoma cells, and further lead to poor prognosis of patients. In addition, lncRNAs have also been found to play a role in the pathogenesis of LUAD (Xu et al., 2021a; Ye et al., 2021). A growing number of studies demonstrate the potential of lncRNAs as biomarkers for various cancers (Xu et al., 2021b; Xie et al., 2021). Yue, N. *et al.* (Yue et al., 2020) found that the high expression of lncRNA pSMG3-AS1 played a carcinogenic role in the development of LUAD. At the same time, recent studies have also shown that it is possible to predict the prognosis of cancer patients by exploring the potential relationship between coding genes and lncRNAs (Wang et al., 2019b; Ye et al., 2022). In this study, we were inspired by the mechanism by which both molecular chaperones and lncRNAs can be involved in the occurrence and development of LUAD. We attempted to construct an independent prognostic model of MCRLncs and explore its value in immune infiltration, immunotherapy effect and prognosis of LUAD.

First, we identified 16 MCRLncs by univariate and multivariate Cox regression analysis. They were AL359513.1, AC004830.2, ‘ZEB1-AS1′, LINC02802, AC026355.2, AC106038.1, AC022034.4, AC093911.1, AC079466.1, AC108136.1, LINC01887, AC019211.1, AL031600.2, AL162632.3, AL024497.2 and LINC00862’. Using these 16 MCRLncs markers, we constructed a risk-prognostic model to predict OS in patients with LUAD. We then divided LUAD patients into high-risk and low-risk groups based on the risk score. KM survival analysis showed that the survival time of LUAD patients in the high-risk group was significantly lower than that of LUAD patients in the low-risk group. LncRNA LINC02802, AC022034.4, AC079466.1, AC108136.1 and AL024497.2 were highly expressed in LUAD in the high-risk group and could be used as predictors for predicting high-risk LUAD. LINC01887, AC019211.1 and AL031600.2 were highly expressed in the high-risk subgroup and low expressed in the low-risk subgroup, indicating that they were protective genes in LUAD patients. We also explored the relationship between clinicopathological factors and OS. Univariate Cox regression analysis showed that tumor stage, T stage, N stage and Riskscore showed statistical differences. However, multivariate Cox regression analysis only found that Riskscore was closely related to the survival time of LUAD patients. This indicates that MCRLncs is an independent prognostic factor in the risk model. The ROC curve and C-index showed that the prognostic model was superior to conventional clinical features in predicting survival in LUAD. We also established a graph that predicted 1 -, 3 -, and 5-year survival in LUAD patients. The calibration curve confirms the good performance of the line chart. KM survival analysis based on clinical variables showed that the clinical outcomes of the high-risk group were significantly worse, which further verified the reliability of our prognostic model. In the same time, the risk model was also confirmed to be significantly associated with sex, age, race, stage I, II, T1, T2, T3, N0, N1, M0 and OS of LUAD patients. The prognosis of LUAD patients is closely related to liver metastases, brain metastases and bone metastases. Unfortunately, there was no statistical significance between the risk model we constructed and the M1 stage.

GO and KEGG pathway enrichment analysis confirmed the Metabolism of xenobiotics by cytochrome P450 and drug metabolism − cytochrome P450 are related to these two metabolic pathways. The genes CYP2F1, CYP2A6, ADH1C, GSTA2, ADH7 and GSTA1 have been shown to be involved in the metabolic pathway regulating the metabolism of cytochrome P450 to xenobiotics. Previous studies have found that the drug-metabolizing enzyme CYP plays an important role in tumor cell progression, which provides new insights into the development of targeted drugs for lung adenocarcinoma (Song et al., 2021b).

Immunotherapy has made great strides in cancer treatment in recent years. Studies have shown that immune checkpoints such as immunosuppressants PD-1 and CTLA4 show important value in inhibiting the occurrence and development of a variety of malignant tumors (Shojaie et al., 2021; Yi et al., 2021). Functional enrichment analysis showed that 16 MCRLncs markers were involved in many immune-related biological processes. To further explore the relationship between MCRLncs and tumor immune microenvironment, we found a positive correlation between the prognostic model and Parainflammation and MHC_class_I pathways by ssGSEA analysis. TMB is the total number of somatic coding mutations. Previous studies have found that higher TMB is associated with OS after immunotherapy for a variety of cancer types, suggesting that TMB can be used as an effective marker of immunotherapy response (Topalian et al., 2016). The prognostic model constructed in this study has no correlation with TMB. In addition, a growing number of studies are using the TIDE score to predict immunotherapy efficacy (Jiang et al., 2018). In our study, the comprehensive score of Exclusion and CAF was higher in the high-risk group. This shows that patients in the high-risk group respond better to immunotherapy, which helps us further screen drugs for LUAD. Next, we discovered bicalutamide, BMS.509,744, Bortezomib, CMK, Docetaxel, Doxorubicin, erlotinib, Gemcitabine, and Obatoclax. Mesylate and Parthenolide as potential therapeutics in the high-risk group through the “pRRophetic” R package. EHT. 1864, Lenalidomide, Methotrexate, PD.0332,991, and Temsirolimus can be used to treat low-risk patients. The IMvigor210 cohort validated the efficacy of immunotherapy agents against target genes. In addition, we found that the stem cell index mRNAsi of LUAD is highly correlated with clinical indicators, indicating that mRNAsi is related to the prognosis of patients.

The pathological stage of the tumor is often used as the basis for clinicians to proceed with the next treatment, because the pathological stage is a decisive factor affecting the prognosis of LUAD (Jurisic et al., 2018). However, studies have shown that LUAD patients at the same pathological stage can have different outcomes (Tan et al., 2021). Therefore, it is necessary to explore new predictive and therapeutic biomarkers to assess the prognosis of patients with LUAD. The MCRLncs model constructed by our study can provide a new approach for the prognosis and immunotherapy of LUAD patients.

Undeniably, our study has certain limitations. Firstly, in our study, we used multiple methods to construct our prognostic model, and our prognostic model was validated to be reliable. However, the clinical data we used to construct the prognostic model were only from the TCGA database. If we tried to validate our prognostic model with an external dataset, we might get different results. Secondly, the prognostic model for the Asian population was not statistically significant. C-index showed that the four clinical variables, age, gender, M, and race, were not well combined with the prognostic model, which may be related to too little sample size. Therefore, we need to recover samples and expand the sample size to improve our prognostic model. Thirdly, the nomogram does not include potential factors such as smoking history, PD-L1, tumor proportion score (TPS), brain metastases, bone metastases, and liver metastases that are associated with the prognosis of patients with LUAD (Yang et al., 2018; Cheng et al., 2019; Wang et al., 2020; Ni et al., 2021; Takamochi et al., 2022). Fourthly, in this study, the IC50 value was used to evaluate drug sensitivity. But in clinical practice, we rarely use parameters to evaluate the efficacy of drugs. Fifthly, we did not clarify the relationship between MCRLncs and tumor-infiltrating immune cells. Finally, we lack clinical follow-up data to validate the value of our prognostic model.

## Conclusion

This study constructed a risk model for 16 MCRLncs in LUAD. This model can effectively predict the OS of LUAD patients. This will help clinicians more accurately identify high-risk patients and further improve the outcome of patients with LUAD. Meanwhile, we transformed LUAD’s risk model into a nomogram prediction model to provide clinicians with a quantitative and convenient prognostic tool and greatly improve the ability of personalized treatment for LUAD patients. We also used an immune checkpoint-based TIDE score to assess the efficacy of immunotherapy. This score predicts sensitivity to certain chemotherapeutic agents and expression of immune checkpoint genes (PD-1/CTLA-4). In addition, risk models play an important role in predicting the immune landscape of LUAD patients. MCRLncs markers can be used as potential therapeutic targets for molecular mechanism studies, which provide an important basis for future studies on the relationship between MCRLncs markers and immunotherapy.

## Data Availability

The original contributions presented in the study are included in the article/Supplementary Material further inquiries can be directed to the corresponding authors.
