# Supplementary figures and images for: Prognostic model and immunotherapy prediction based on molecular chaperone-related lncRNAs in lung adenocarcinoma
Source: Front Genet. 2022 Oct 13;13:975905. doi: 10.3389/fgene.2022.975905 (PMC9606628; doi:10.3389/fgene.2022.975905)

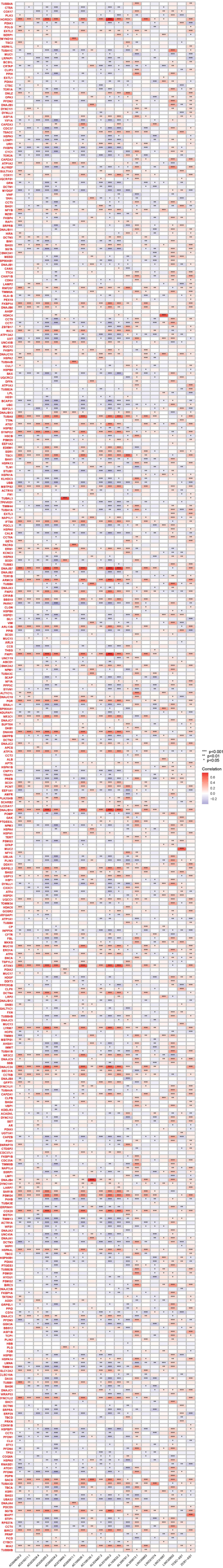

Supplement: Supplementary file 1 [file Image1.JPEG]
