# Supplementary material for: Prognostic model and immunotherapy prediction based on molecular chaperone-related lncRNAs in lung adenocarcinoma
Source: Front Genet. 2022 Oct 13;13:975905. doi: 10.3389/fgene.2022.975905 (PMC9606628; doi:10.3389/fgene.2022.975905)
Supplement: Supplementary file 2 [file Table1.DOC]

**Table S1: 17 GSEA functional enrichment pathways based on molecular signiture database**

| Standard name | Systematic name | Brief description | Gene members | External links |
| --- | --- | --- | --- | --- |
| GO_CHAPERONE_BINDING | M18579 | Interacting selectively and non-covalently with a chaperone protein, a class of proteins that bind to nascent or unfolded polypeptides and ensure correct folding or transport. [PMID:10585443] | 104 | <http://amigo.geneontology.org/amigo/term/GO:0051087> |
| GO_CHAPERONE_COFACTOR_DEPENDENT_PROTEIN_REFOLDING | M23942 | The process of assisting in the correct posttranslational noncovalent assembly of proteins, which is dependent on additional protein cofactors. This process occurs over one or several cycles of nucleotide hydrolysis-dependent binding and release. [GOC:rb] | 32 | <http://amigo.geneontology.org/amigo/term/GO:0051085> |
| GO_CHAPERONE_COMPLEX | M26029 | A protein complex required for the non-covalent folding or unfolding, maturation, stabilization or assembly or disassembly of macromolecular structures. Usually active during or immediately after completion of translation. Many chaperone complexes contain heat shock proteins. [GOC:bhm, PMID:21855797] | 25 | <http://amigo.geneontology.org/amigo/term/GO:0101031> |
| GO_CHAPERONE_MEDIATED_AUTOPHAGY | M24303 | The autophagy process which begins when chaperones and co-chaperones recognize a target motif and unfold the substrate protein. The proteins are then transported to the lysosome where they are degraded. [GOC:pad, GOC:PARL, PMID:22743996, PMID:23434281] | 16 | <http://amigo.geneontology.org/amigo/term/GO:0061684> |
| GO_CHAPERONE_MEDIATED_PROTEIN_COMPLEX_ASSEMBLY | M11479 | The aggregation, arrangement and bonding together of a set of components to form a protein complex, mediated by chaperone molecules that do not form part of the finished complex. [GOC:ai] | 22 | <http://amigo.geneontology.org/amigo/term/GO:0051131> |
| GO_CHAPERONE_MEDIATED_PROTEIN_FOLDING | M16577 | The process of inhibiting aggregation and assisting in the covalent and noncovalent assembly of single chain polypeptides or multisubunit complexes into the correct tertiary structure that is dependent on interaction with a chaperone. [GOC:dph, GOC:vw] | 60 | <http://amigo.geneontology.org/amigo/term/GO:0061077> |
| GO_CHAPERONE_MEDIATED_PROTEIN_TRANSPORT | M24579 | The directed movement of proteins into, out of or within a cell, or between cells, mediated by chaperone molecules that bind to the transported proteins. [GOC:mah, PMID:20378773] | 11 | <http://amigo.geneontology.org/amigo/term/GO:0072321> |
| GO_ENDOPLASMIC_RETICULUM_CHAPERONE_COMPLEX | M17527 | A protein complex that is located in the endoplasmic reticulum and is composed of chaperone proteins, including BiP, GRP94; CaBP1, protein disulfide isomerase (PDI), ERdj3, cyclophilin B, ERp72, GRP170, UDP-glucosyltransferase, and SDF2-L1. | 10 | <http://amigo.geneontology.org/amigo/term/GO:0034663> |
| GO_PROTEIN_FOLDING_CHAPERONE | M26670 | Interacting selectively and non-covalently with any protein or protein complex (a complex of two or more proteins that may include other nonprotein molecules) that contributes to the process of protein folding. [GOC:mtg_cambridge_2009] | 27 | <http://amigo.geneontology.org/amigo/term/GO:0044183> |
| GO_REGULATION_OF_CHAPERONE_MEDIATED_AUTOPHAGY | M25277 | Any process that modulates the frequency, rate or extent of chaperone-mediated autophagy. [GO_REF:0000058, GOC:pad, GOC:PARL, GOC:TermGenie, PMID:20176123] | 8 | <http://amigo.geneontology.org/amigo/term/GO:1904714> |
| GO_REGULATION_OF_CHAPERONE_MEDIATED_PROTEIN_FOLDING | M29354 | Any process that modulates the frequency, rate or extent of chaperone-mediated protein folding. [GO_REF:0000058, GOC:TermGenie, PMID:24375412] | 5 | <http://amigo.geneontology.org/amigo/term/GO:1903644> |
| REACTOME_ACTIVATION_OF_CHAPERONE_GENES_BY_XBP1S | M790 | Genes involved in Activation of Chaperone Genes by XBP1(S)  XBP1(S) | 46 | <http://www.reactome.org/cgi-bin/eventbrowser_st_id?ST_ID=REACT_18273> |
| REACTOME_ATF6_ATF6_ALPHA_ACTIVATES_CHAPERONE_GENES | M801 | ATF6 (ATF6-alpha) activates chaperone genes | 10 | <https://www.reactome.org/content/detail/R-HSA-381183> |
| REACTOME_ATF6_ATF6_ALPHA_ACTIVATES_CHAPERONES | M794 | ATF6 (ATF6-alpha) activates chaperones | 12 | <https://www.reactome.org/content/detail/R-HSA-381033> |
| REACTOME_CHAPERONE_MEDIATED_AUTOPHAGY | M27937 | Chaperone Mediated Autophagy | 22 | <https://www.reactome.org/content/detail/R-HSA-9613829> |
| REACTOME_HSP90_CHAPERONE_CYCLE_FOR_STEROID_HORMONE_RECEPTORS_SHR_ | M27251 | HSP90 chaperone cycle for steroid hormone receptors (SHR) | 57 | <https://www.reactome.org/content/detail/R-HSA-3371497> |
| REACTOME_IRE1ALPHA_ACTIVATES_CHAPERONES | M27282 | IRE1alpha activates chaperones | 50 | <https://www.reactome.org/content/detail/R-HSA-381070> |

**Table S2: Multivariate cox regression analysis of twenty prognostic lncRNAs**

| LncRNAs | Coef | HR | HR.95L | HR.95H | pvalue |
| --- | --- | --- | --- | --- | --- |
| AL359513.1 | -0.356382264 | 0.700204906 | 0.497914665 | 0.984680597 | 0.040490368 |
| AC004830.2 | 0.765500787 | 2.150070833 | 1.285128084 | 3.597154746 | 0.003553092 |
| `ZEB1-AS1` | 0.400819851 | 1.493048273 | 1.017467531 | 2.190923126 | 0.040514523 |
| LINC02802 | 0.557295611 | 1.745944397 | 1.333296993 | 2.286303693 | 0.000051 |
| AC026355.2 | -0.344343703 | 0.708685309 | 0.55503845 | 0.904865 | 0.005749187 |
| AC106038.1 | 2.406357366 | 11.09347798 | 1.553643276 | 79.21075289 | 0.016427853 |
| AC022034.4 | 0.561960968 | 1.754108881 | 1.124324769 | 2.736662973 | 0.013273634 |
| AC093911.1 | -1.426514608 | 0.240144463 | 0.069076259 | 0.834865179 | 0.024841342 |
| AC079466.1 | 0.766722602 | 2.152699427 | 1.57522815 | 2.941868975 | 0.0000015 |
| AC108136.1 | 0.382419817 | 1.465827336 | 1.059462147 | 2.028057145 | 0.020961925 |
| AC060234.2 | -0.889343682 | 0.410925362 | 0.148104501 | 1.140138564 | 0.087622031 |
| LINC01374 | -0.450426398 | 0.637356326 | 0.348828406 | 1.164535569 | 0.143014822 |
| LINC01887 | -1.286673156 | 0.276188091 | 0.083013584 | 0.918884096 | 0.03591443 |
| AC019211.1 | -1.485284648 | 0.226437876 | 0.065623837 | 0.781333647 | 0.018750819 |
| AL031600.2 | -1.241791004 | 0.288866393 | 0.121793904 | 0.685122903 | 0.004829853 |
| AL162632.3 | 1.835026028 | 6.265297218 | 2.192615325 | 17.90279798 | 0.000613569 |
| AL024497.2 | 0.439936192 | 1.552608147 | 1.205487191 | 1.999682848 | 0.00065577 |
| LINC00862 | 0.362596774 | 1.437056283 | 1.060680028 | 1.946987506 | 0.019275219 |
| AL358781.2 | -0.473924591 | 0.622554197 | 0.382248766 | 1.01393062 | 0.056861643 |
| `TESC-AS1` | 0.277935921 | 1.320401585 | 0.94195615 | 1.850893318 | 0.106756144 |

**Table S3: Multivariable Cox proportional hazards analyses**

| items | HR | HR.95L | HR.95H | P-value |
| --- | --- | --- | --- | --- |
| age | 0.994077365690332 | 0.973391352050761 | 1.01520298787933 | 0.579816651876381 |
| gender | 0.92354271011912 | 0.612868409739303 | 1.39170354330546 | 0.703824701628473 |
| race | 1.36392907157469 | 0.742479874589411 | 2.50552584110826 | 0.317164038513405 |
| stage | 1.62286343919191 | 0.962771551032703 | 2.73552509880544 | 0.0691330914060838 |
| T | 1.13345644892412 | 0.861177239794524 | 1.4918224289279 | 0.371472893509474 |
| M | 0.505822363970863 | 0.139530528390545 | 1.83369379335348 | 0.299628173646606 |
| N | 1.25097698488649 | 0.782723839013749 | 1.99935576088697 | 0.349279725682246 |
| riskScore | 1.01114132550378 | 1.00655483790036 | 1.01574871198697 | 1.78257322370858E-06 |

**Figure S1.** Heatmaps of associations between 20 prognostic MCRLncs and 417 molecular chaperone genes in a risk model. *p < 0.05，**p < 0.01，***p < 0.001.
